# *Dendropanax morbifera* Extracts for Cosmetic Applications: Systematic Review and Meta-Analysis

**DOI:** 10.3390/cimb46120808

**Published:** 2024-11-25

**Authors:** Le Thi Nhu Ngoc, Ju-Young Moon, Young-Chul Lee

**Affiliations:** 1 Department of Nano Science and Technology Convergence, Gachon University, 1342 Seongnam-Daero, Sujeong-gu, Seongnam-si 13120, Gyeonggi-do, Republic of Korea; nhungocle92@gmail.com; 2 Major in Beauty Convergence, Kwangwoon University, 20 Kwangwoon-ro, Nowon-gu, Seoul 01897, Republic of Korea; bora7033@naver.com; 3 Department of BioNano Technology, Gachon University, 1342 Seongnam-daero, Sujeong-gu, Seongnam-si 13120, Gyeonggi-do, Republic of Korea

**Keywords:** *Dendorpanax morbifera*, hyperpigmentation, anti-inflammatory, antimicrobial, meta-analysis

## Abstract

We have conducted a systematic review and meta-analysis to evaluate the cosmetic applications of *Dendropanax morbifera* extracts (DMEs). A total of 261 articles were screened; however, after eliminating inappropriate studies, only 16 individual studies were eligible. The comparative standardized mean difference (SMD) between the DME treatment and control groups was used to evaluate the cosmetic properties of DME, including its biocompatibility, whitening effects, and anti-inflammatory and antimicrobial properties. DME treatment exhibited positive results in controlling hyperpigmentation, including effective inhibition of the production of tyrosinase and melanin, with SMDs of 6.85 [4.27, 9.44] and 23.38 [12.94, 33.82], respectively. Moreover, the results confirmed the anti-inflammatory properties in terms of suppressing the expression of interleukin markers (ILs) (SMD = 5.22 [3.12, 7.33]) and reducing NO production (SMD = 6.92 [2.89, 10.96]). DME treatment also effectively inhibited bacteria growth, which causes skin disorders. According to the results, DMEs are shown to be highly biocompatibility, with excellent anti-hyperpigmentation, anti-inflammatory, and antimicrobial properties that contribute significantly to improving skin appearance. The findings provide strong evidence for further research into the in vivo effects of DMEs and their potential cosmetic applications, which could lead to clinical trials in the future.

## 1. Introduction

The use of botanical extracts for therapeutic purposes, such as skincare and cosmetics, has gained popularity in recent times. One of the promising candidates is the *Dendropanax* genus of the Araliaceae family, which has been extensively used in Korea, Japan, South America, and other parts of the world [1,2]. There are approximately 6 to 10 species in this genus, including *D. morbifera*, *D. gonatopodus*, *D. dentiger*, *D. capillaris*, *D. chevalieri*, and *D. arboreus*. In Korea, *D. morbifera* is native to the tropics and is predominantly found on Jeju Island [2,3,4,5]. The Ministry of Food and Drug Safety, Korea Food and Drug Administration have registered various parts of the *D. morbifera* plant, including the edible leaf, stems, seeds, bark, and roots, as food additives and alternative folkloric medicine [2].

*D. morbifera* has various bioactive compounds, both in pure form and crude extracts, demonstrating effectiveness in their biological activities. These compounds are categorized as polyphenols, flavonoids, tannins, pyrimidines, essential oils, terpenoids, phenol carboxylic acids, and alkaloids, which can be applied in medical and cosmetic fields [2,6,7,8,9]. Notably, almost all of these bioactive compounds show antioxidant, anti-inflammatory, anti-cancer, and neuroprotective properties. Some of the most notable compounds are quercetin, rutin, gallic acid, 2,5-dihydroxybenzoic acid, catechin, 4-hydroxybenzoic acid, caffeic acid, syringic acid, p-coumaric acid, trans-ferulic acid, salicylic acid, hesperidin, naringin, resveratrol, myricetin, and trans-cinnamic acid [2].

As the demand for natural and sustainable ingredients in cosmetic products increases, exploring the scientific basis behind the cosmetic and dermatology efficacy of *D. morbifera* extract (DME) becomes imperative. Recent studies have proven the cosmetic properties of DME, such as antioxidant, anti-inflammatory, antimicrobial, moisturizing, and biocompatibility ones. The DME possesses potent antioxidant properties that are functional for skin-whitening and skin-aging control. The antioxidant properties are demonstrated by high scavenging activities for 1,1-diphenyl-2-picrylhydrazyl (DPPH) and 2,2′-azino-bus(3-ethylbenzothiazoline-6-sulfonic acid (ABTS), reactive oxygen species (ROS) scavenging activities (60%–92%), superoxide dismutase (SOD)-like activities, and low cytotoxicity, owing to their high contents of flavonoid and phenolic compounds [10,11,12,13,14]. Additionally, the extracts show potential anti-inflammatory properties by reducing the production of NO, inducible NO synthase and interleukin markers (ILs), nuclear translocation of nuclear factor- κ B (NF- κ B), and tumor necrosis factor- α (TNF- α ) in lipopolysaccharide (LPS)-stimulated RAW264.7 cells [15]. The extract can also mitigate 2,4-dinitrochlorobenzene (DNCB)-induced inflammatory dermatitis in vivo without side effects, suggesting that it can be used for development as a botanical drug to treat atopic dermatitis based on its anti-inflammatory activities [15]. The DME also exhibits antimicrobial activity against *Streptococcus mutans*, *Candida albicans*, *Staphylococcus aureus*, *Staphylococcus epidermidis*, *Escherichia coli*, and *Pseudomonas aeruginosa* [4,16,17]. For instance, the hot water and ethanol extracts from the leaves of *D. morbifera* significantly eliminate *Porphyromonas gingivalis* after 6 h of incubation, with the minimum inhibitory concentration (MIC) being around 3.13 and 6.25 mg/mL, respectively [18]. The fermented extract of boughs of *D. morbifera* (200 mg/mL) has strong antimicrobial effects against *S. epidermidis* and *S. aureus* with inhibition zone diameters of about 9.3–15.2 mm, suggesting that the extract produced from *D. morbifera* bough has the potential to produce health-oriented food materials [19]. The 60% ethanol extract of roasted *D. morbifera* leaves shows the ability to inhibit the growth of *Bacillus cereus*, *S. aureus*, and *Pichia jadinii* with an MIC higher than 250 μg/mL [20]. Moreover, they can inhibit tyrosinase activity and melanin formation in α -melanocyte-stimulating hormone ( α -MSH)-induced melanoma B16/F1 cells, indicating whitening effects [13,14,21]. They also have high promotive effects in growth factor IGF protein expression (156%) and strongly inhibit TGF β 2 protein expression (78.3%) in human hair cells, making them beneficial for hair growth applications [12]. In addition, 1-tetradecanol isolated from *D. morbifera* has moisturizing properties that can aid in preventing hair loss and enhancing hair density. The study examined the skin moisturizing effect using HR-1 hairless mice. The transepidermal water loss (TEWL) in the group treated with 1-tetradecanol was significantly lower than that of the control group, showing a reduction of 30% [5].

Although the application of DME to cosmetics has attracted the attention of scientists, no systematic review has addressed this interesting topic. Therefore, this paper presents a comprehensive systematic review and meta-analysis of the existing literature, aiming to elucidate the effectiveness of DME in cosmetic applications. By analyzing and synthesizing findings from the electronic literature, this study provides a comprehensive understanding of the beneficial effects of DME on different skin conditions, such as hyperpigmentation, inflammation, and microbial infections. By integrating diverse articles, it aims to provide valuable insights into the efficacy of DME in cosmetic applications. The findings of this review may guide future research, inform product development in the cosmetics industry, and provide a solid foundation for evidence-based decisions regarding the use of DME in skin care formulations.

## 2. Materials and Methods

### 2.1. Literature Search Strategy

This systematic review was designed according to the “Preferred Reporting Items for Systematic Reviews and Meta-Analysis” (PRISMA) 2009 protocol. A literature search for the application of DME in cosmetic products was performed to obtain relevant papers published up to 2024. Data from Korean and English databases were collected from the Cochrane Library, PubMed, Elsevier, EMBASE, and MedRxiv. An electronic search was performed using the following keywords: *D. morbifera*, DME, antioxidants, anti-inflammatory, antimicrobial, whitening effects, and cosmetic benefits. This systematic review has been registered on PROSPERO under the registration number CRD42024506153.

### 2.2. Study Selection and Data Extraction

#### 2.2.1. Study Selection

This review included all in vitro studies that investigated the application of DME to improve skin appearance, specifically focusing on anti-aging, whitening effects, and acne treatments. In vitro studies with at least one matched perspective between the control and treatment groups were considered. Individual studies with multiple purposes were selected if they directly compared the treatment and the control groups. The main outcomes were presented as the mean ± standard difference (SD). There were no restrictions on the treatment and control groups, raw materials, extraction methods, or geographical regions. All articles were published in Korean or English and were translated into English for easy data extraction.

Studies on DME applications in other fields, such as medicine and the food industry, were not included. Articles that were partially or entirely duplicated from different studies were also excluded. This study did not include conference abstracts, reviews, primary manuscripts, and seminar presentations.

#### 2.2.2. Data Extraction

The selected studies were screened by three independent reviewers to extract the appropriate data for the systematic review and meta-analysis. The extraction data include the first author’s name, region of study, year of publication, raw materials (stem, leave, branch, and root), extraction method, study design, cosmetic properties (antioxidant, anti-inflammatory, antimicrobial, cell viability, and whitening effect), and primary outcomes (mean ± SD values).

### 2.3. Meta-Analysis

For all continuous outcomes, the mean and SD of each primary outcome in both the control and DME treatment groups were pooled using the random-effects model and calculated as the SMD (95% confidence interval CI) [22]. The SMD was statistically significant at the 5% level (*p* < 0.05) if the 0 value was not within the 95% CI. SMDs are categorized into three phases: small (SMD ≤ 0.2), moderate (SMD approximately 0.5), and large (SMD ≥ 0.8) [22]. The heterogeneity of the analysis was quantified using the I^2^ statistics. Heterogeneity was considered small, moderate, or large when it was less than 25%, approximately 25–50%, or >50%, respectively [22]. The bias was assessed using a funnel plot.

All analyses were performed using Review Manager (version 5.3, Copenhagen: The Nordic Cochrane Centre, The Cochrane Collaboration, 2014) [23].

## 3. Results

### 3.1. Characteristics of Included Studies

**Figure 1:**
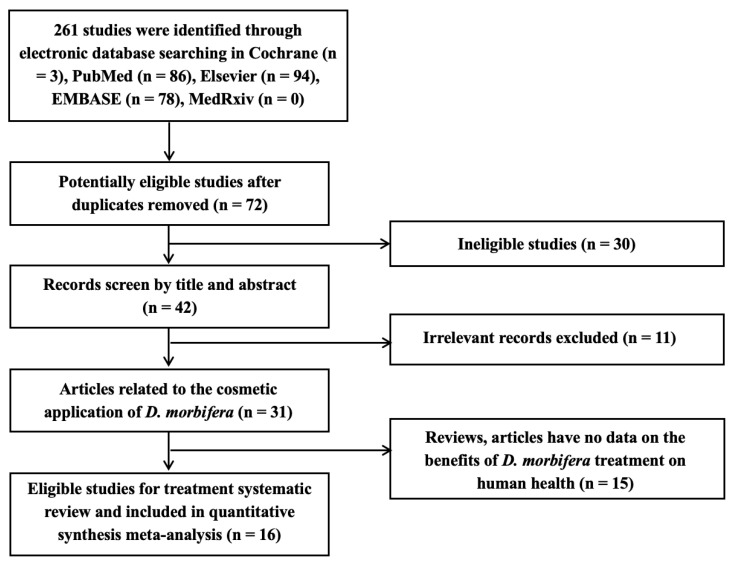
Systematic screening stages of the literature review on *D. morbifera* extracts (DMEs).

The screening process for the summarized articles is shown in the flowchart (Figure 1). In this study, 261 publications were collected by searching electronic databases using the aforementioned keywords. After the careful screening of these articles by the three reviewers, individual studies were selected to assess the cosmetic properties of DME using a meta-analysis.

#### Description of Included Studies

The main characteristics of the 16 included studies are summarized in Table 1. Four studies reported the anti-inflammation effects of DME in skin treatment [12,15,24,25]. Four studies investigated the antimicrobial properties of DME [4,16,19,26]. Seven studies demonstrated the whitening effects of DME for improving skin appearance [3,5,13,14,21,27,28]. The biocompatibility of the extracts with human skin and hair cell lines was investigated in five studies [4,12,13,14,29]. All the included studies were conducted only in Korea between 2010–2024, since *D. morbifera* is cultivated primarily in Korea. Various parts of *D. morbifera*, including the leaves (n =15), wood (n = 1), stems (n = 1), and branches (n = 3), were used in the studies. *D. morbifera* underwent various extraction methods, such as methanol, ethanol, hot water extraction, and microwave-assisted extraction, before being tested in vitro on human cell lines and bacteria. Most studies reported high antioxidant compounds (flavonoids and polyphenolics), suggesting high biocompatibility properties. The studies included three major experimental subject types: normal human cells (HFDPC and HaCaT cells), cancer cells (RAW264.7, L929, B16F10, and EL-4 T), and bacteria (*S. aureus*, *S. mutans*, and *P. acnes*). The extracted data from several main outcomes, such as cell viability (%), inhibition of the expression of pro-inflammatory markers (IL and TNF- α ) (pg/mL), NO, inhibition of bacterial growth (%), and inhibition of melanin, tyrosinase, and elastase productions (%), were used in the meta-analysis to increase the statistical significance of the results.

### 3.2. Quality Assessment of Included Studies

**Figure 2:**
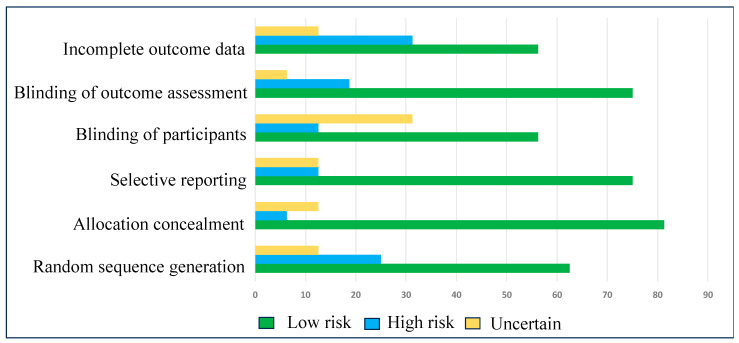
Risk of bias in individual studies.

Eligible studies were qualified by considering the bias in the random sequence generation, selective reporting, allocation concealment, blinding of participants, blinding of outcome assessment, and incomplete outcome data, following the Cochrane guidelines [23]. There were three levels of assessment–low risk, high risk, and uncertain–to exhibit the lack of information and uncertainty over the potential for bias. Nearly all the criteria exhibited a low risk of bias, resulting in an evident enhancement of the statistical significance of the meta-analysis (Table 2 and Figure 2).

### 3.3. Beneficial Effects of D. morbifera in Cosmetic Applications

#### 3.3.1. Biocompatibility

**Figure 3:**
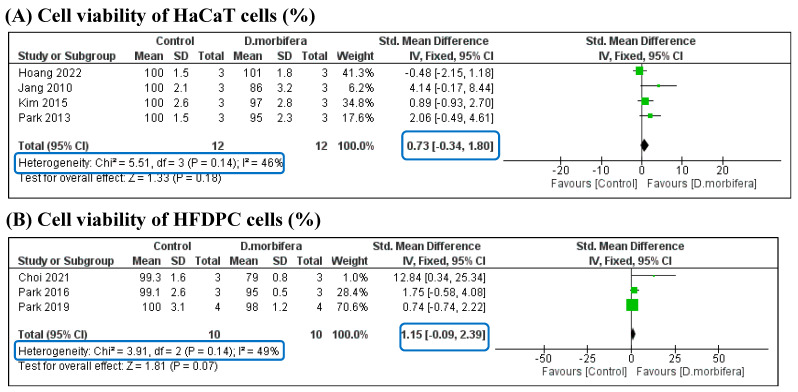
Comparison of the cell viability (%) of HaCaT (**A**) and HFDPC (**B**) cells between the control and the DME treatment groups [4,12,13,14,16,26,29].

Two types of human cells are commonly used in in vitro experiments for cosmetic applications: normal human skin (HaCaT) cells and normal human follicle dermal papilla (HFDPC) cells. In this study, the biocompatibility of DME was analyzed in two comparisons based on the treatment of the two cell lines (Figure 3 and Table 3). The cytotoxicity of DME on HaCaT and HFDPC cells was compared with that of the control group. The pooled SMD (0.80 [−0.30, 1.90] and 1.15 [−0.09, 2.39], respectively) indicated that there was no difference in the viability of HaCaT and HFDPC cells between the two groups, suggesting that DME is highly biocompatible with human skin fibroblast.

#### 3.3.2. Whitening Effects

**Figure 4:**
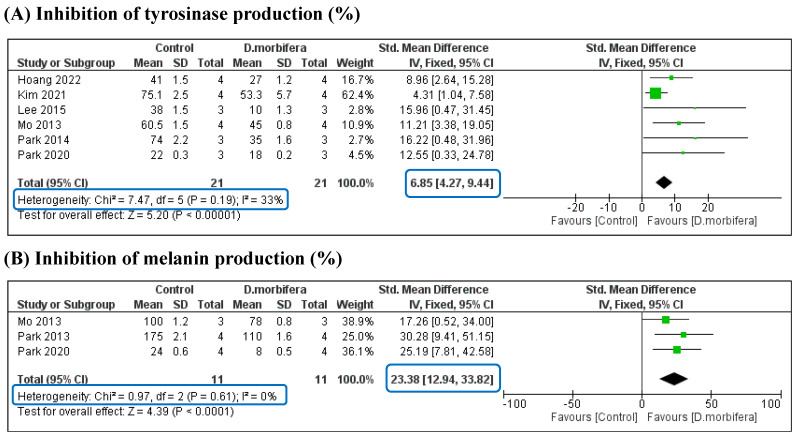
Comparison of downregulating the synthesis of tyrosinase (**A**) and melanin (**B**) between the control and the DME treatment groups [3,5,13,14,21,27,28].

Three comparisons were performed between the mean differences of the control and the DME treatment groups from three perspectives: inhibition of tyrosinase, melanin, and elastase production (%). The results of each study showed that the synthesis of tyrosinase and melanin in the control group was significantly higher than that in the DME treatment group. Meta-analysis confirmed that there were significant differences in the levels of tyrosinase and melanin between the two groups, with SMDs of 6.85 [4.27, 9.44] and 23.38 [12.94, 33.82], respectively (Figure 4 and Table 3). In addition, the analyses were highly significant because of the low heterogeneity of these comparisons (I^2^ = 0% and 33%). The significant downregulation of tyrosinase and melanin suggests that DMEs are potential ingredients for whitening cosmetic formulations.

#### 3.3.3. Anti-Inflammation Properties

**Figure 5:**
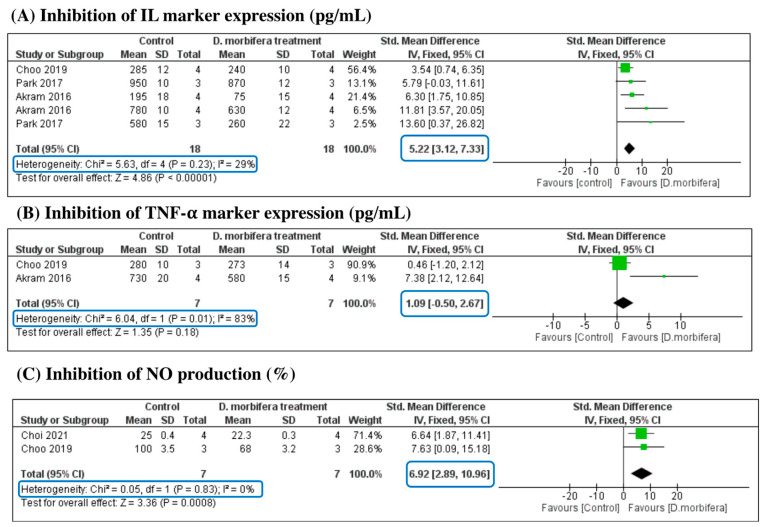
Comparison of inhibition of the expression of pro-inflammatory markers (IL (**A**) and TNF- α (**B**)) and the production of NO (**C**) between control and DME treatment groups [15,24,25,26].

This analysis aimed to evaluate the impact of DME on inflammation signals based on a comparison with three sub-criteria: inhibition of the expression of IL markers (pg/mL), inhibition of the expression of TNF-α (pg/mL), and inhibition of NO production (%). The SMD of each analysis is presented in Figure 5 and Table 3. The meta-analysis indicated that the DME treatment significantly inhibited the expression of pro-inflammatory IL markers, with an SMD of 5.22 [3.12, 7.33] (I^2^ = 29% and *p* = 0.23). The production of NO was also reduced in the treatment group compared to the control groups, with significant differences in SMD (6.92 [2.89, 10.96], I^2^ = 0%, and *p* = 0.83). However, the expression of TNF- α was not significantly different between the two groups, indicating that the DME treatment was ineffective in suppressing the pro-inflammatory TNF-α marker (SMD = 1.09 [−0.5, 2.67]).

#### 3.3.4. Antimicrobial Properties

**Figure 6:**
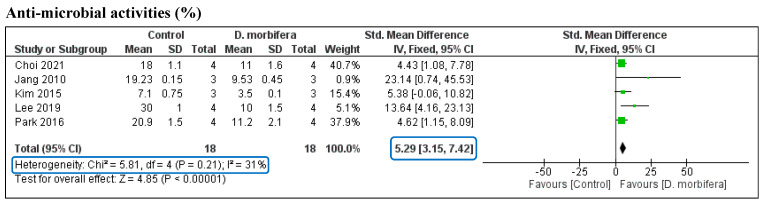
Comparison of bacterial growth inhibition between the control and the DME treatment groups [4,12,16,17,26].

The pooled SMD of the five studies on the antimicrobial activity of DME was assessed by analyzing the statistical differences in bacterial growth between the treatment and the control groups. The pooled SMD was significantly different between the two groups (SMD = 5.29 [3.15, 7.42]) without any heterogeneity (I^2^ = 31%, *p* = 0.21) (Figure 6 and Table 3). This indicates that DMEs can effectively suppress bacterial growth and therefore be used in the treatment of skin disorders caused by microbes.

#### 3.3.5. Subgroup Analysis

Due to the different extraction methods used in these included studies (e.g., methanol, ethanol, water, n-hexane, and microwave-assisted extractions), subgroup analyses were designed to evaluate the beneficial effects of DME in improving skin appearance. However, due to the limited number of included studies, subgroup analyses were performed only on methanol and ethanol extractions, which could show statistical significance outcomes when comparing the control and treatment groups.

**Figure 7:**
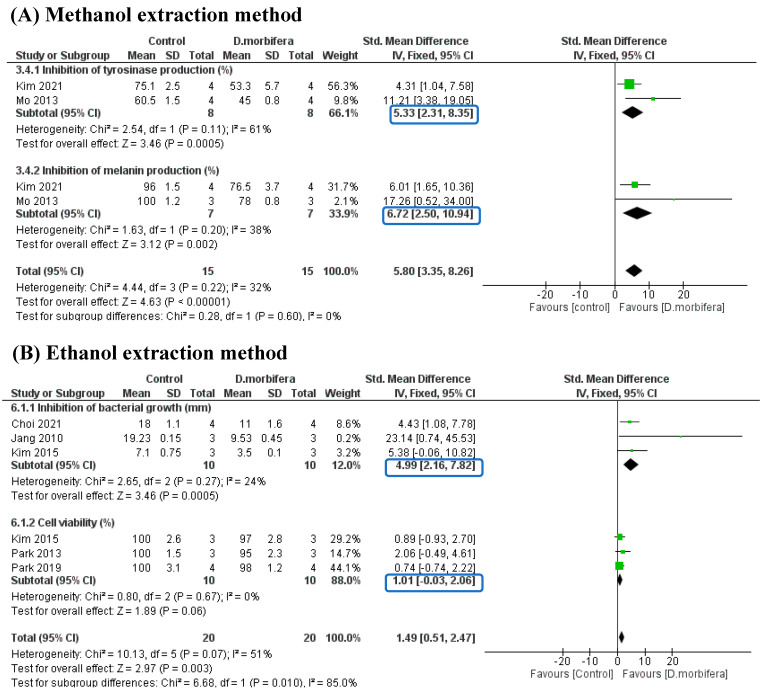
Subgroup analysis evaluating the efficacy of DME-based methanol extraction [3,27] (**A**) and DME-based ethanol extraction when compared to the control group [4,14,16,26,29] (**B**).

The first subgroup analysis evaluated the efficacy of DME-based methanol extraction in reducing the production of tyrosinase and melanin. Two eligible studies were included in this manner [3,27], which showed significant differences in tyrosinase and melanin production between the two groups. The DME treatment resulted in a decrease in tyrosine and melanin content compared to the control group. This subgroup achieved good statistical significance, with I^2^ = 32% and *p* = 0.22 (Figure 7A).

In the second subgroup analysis, the ethanol extraction method was used, which was divided into two subcomparisons (Figure 7B). The first subcomparison evaluated the inhibition of bacterial growth (mm), while the second subcomparison addressed cell viability (%). The results showed that bacterial growth was significantly decreased in the DME treatment group compared to the control group (SMD = 4.99 [2.16, 7.82], I^2^ =24%, and *p* = 0.27). The cell viability comparison showed no significant difference between the DME and the control group (SMD = 1.01 [−0.03, 2.06] and I^2^ = 0%), suggesting that DME treatment had a low toxicity and did not affect the cell proliferation of HaCaT and HFDPC cells. This subgroup analysis indicated that the DME treatment helped eliminate harmful factors for the skin, such as bacteria with a high biocompatibility.

## 4. Analysis Bias

**Figure 8:**
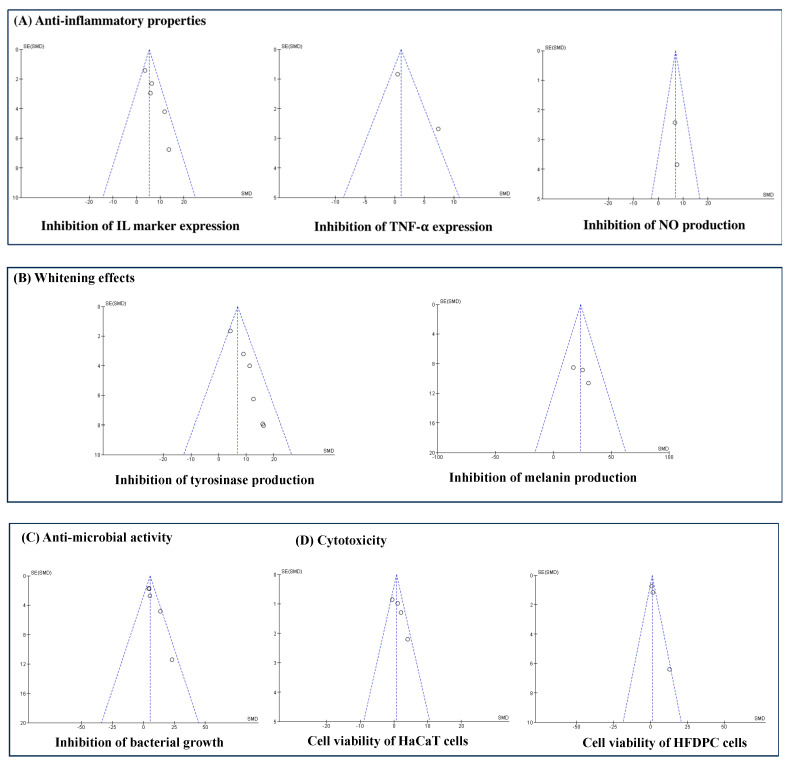
Publication bias of the included studies on the effectiveness of using DME in cosmetic applications.

Funnel plots were constructed to detect publication biases. The results indicated that the population of the studies included for each analysis criterion was small, which led to underrated meta-analysis results and publication biases (Figure 8). Therefore, a meta-analysis with numerous prospective studies should be performed to improve the statistical significance and publication bias.

## 5. Discussion

We conducted a systematic review and meta-analysis of 16 in vitro studies to evaluate the cosmetic properties of DMEs. This study found that DME, despite using different extraction methods, contained high flavonoid and phenolic contents, which indicated a high antioxidant activity. The biocompatibility of DMEs was confirmed through a meta-analysis. Treatment with these extracts did not result in significant toxicity to normal human skin or human hair follicle cells compared with the control group. The SMDs between the two groups for HaCaT and HFDPC cells were 0.80 [−0.30, 1.90] (I^2^ = 47%) and 1.15 [−0.09, 2.39] (I^2^ = 49%), respectively. These extracts possess potent antioxidant properties that can effectively eliminate the production of ROS, which causes oxidative stress in cell lines, ultimately leading to improved cell proliferation. Considering these properties, this study suggests that these extracts can be safely used in cosmetic applications.

Hyperpigmentation is a skin condition in which specific areas of the skin become darker or have pigmentation [30]. There are different types of this condition, including freckles, sunspots, age spots, post-inflammatory hyperpigmentation (PIH), and melasma. Darkening occurs owing to excess melanin and tyrosinase production [31]. Melanin is synthesized by melanocytes and other specialized cells in the basal layer of the epidermis [30]. For instance, long-term exposure to UV rays leads to more melanin production as a protective response, causing tanning, uneven pigmentation, and dark spot formation. Tyrosinase, an enzyme that catalyzes the conversion of tyrosine to dihydroxyphenylalanine (DOPA), is the initial step of melanin synthesis [31]. DOPA is then converted into dopachrome, which is either pheomelanin or eumelanin. Pheomelanin contributes to red and yellow pigments, while eumelanin produces black and brown pigments. Once synthesized, melanin is transported to melanosomes and transferred to neighboring keratinocytes in the epidermis, thus determining skin color [31]. Therefore, treatment for hyperpigmentation should contain ingredients that inhibit melanin and tyrosinase production to promote skin tone. Hydroquinone, kojic acid, arbutin, and plant extracts inhibit tyrosinase, reduce melanin production, and lighten dark spots [32]. DMEs contain antioxidant compounds which can help reduce skin oxidative stress. This indirectly inhibits melanin and tyrosinase activity, which prevents melanin overproduction and in turn reduces hyperpigmentation [14,31]. Antioxidants also help neutralize free radicals that contribute to skin damage and hyperpigmentation and reduce lipid peroxidation, oxidative stress and various oxidative insults, preventing melanin overproduction [3,5,32]. DME has been shown to have a higher DPPH-radical scavenging activity (82.92 ± 0.49%) than butylated hydroxytoluene (56.71 ± 6.34%) and one that is at the same level as vitamin C (90.11 ± 0.13%), at the highest concentration of 500 μg/mL [4]. Furthermore, the 95% ethanol extractions of *D. morbifera* (roots, leaves, and stems) in the range of 10–200 μg/mL effectively inhibit intracellular ROS production against hepatocellular injury induced by *t*-BHP in HepG2 cells [33]. Notably, DMEs have shown the potential to downregulate tyrosinase expression, which results in a decrease in tyrosinase production. These extracts can help prevent the uneven distribution of hyperpigmentation-associated pigmentation by modulating melanin transfer to neighboring skin cells, thereby influencing the transport and distribution of melanin within the skin [3,5,32]. Moreover, DME can promote collagen synthesis, which supports the skin and contributes to overall skin health [3]. Various in vitro studies have investigated the intracellular mechanisms. It has been found that DME inhibits α-melanocyte stimulating hormone (α-MSH)-stimulated intra/extracellular melanogenesis on melanoma B16/F1 cells in a concentration-dependent manner [14]. The ethyl acetate and aglycone fractions of *D. morbifera* leaf extract are found to be more effective when inhibiting α-MSH (21% and 44% at 25 μg/mL, respectively) than arbutin (15% at 25 μg/mL), a whitening agent [14]. The extraction of *D. morbifera* leaf has been reported to decrease melanin content by inhibiting the intracellular tyrosinase activity and protein expression of tyrosinase and tyrosinase-related protein-2 (TRP-2) at concentrations of 25 and 50 μg/mL [28]. Another study shows that *D. morbifera* stem extract has high collagenase and elastase inhibitory activities (79.5% and 23%), with *D. morbifera* leaf extract exhibiting potential tyrosinase inhibitory activity (53.3%) [3]. In addition, inflammation can contribute to post-inflammatory hyperpigmentation [34], and the compounds found in DME may exhibit anti-inflammatory effects, potentially reducing skin inflammation and subsequent skin darkening. Through the meta-analysis, it is confirmed that DME significantly downregulates tyrosinase and melanin (SMD of 6.85 [4.27, 9.44] and 23.38 [12.94, 33.82], respectively), suggesting that DMEs are potential whitening ingredients.

Inflammation is a natural biological response to injury, irritation, infection, or damage. Inflammation plays a significant role in various skin disorders [35]. While acute inflammation is a protective response to injury or infection, chronic inflammation can develop and worsen certain conditions, such as acne, psoriasis, eczema, and rosacea [35]. Inflammation is critical in developing inflammatory acne lesions (e.g., pimples, blackheads, and whiteheads). The immune system responds to bacteria in the hair follicles, leading to redness and swelling. Psoriasis is a skin condition in which immune cells are activated, causing them to multiply faster than usual [36]. Rosacea is a chronic skin condition that primarily affects the face, causing redness, visible blood vessels, and sometimes red bumps. Inflammation and an overactive immune response are believed to contribute to the development of rosacea [37]. Moreover, inflammation can contribute to changes in skin color through various mechanisms. Post-inflammatory hyperpigmentation occurs when the skin darkens due to the healing process after an inflammatory event [34]. Inflammation increases melanin production and causes hyperpigmentation. Therefore, it is crucial to understand the intracellular mechanisms of inflammation during the treatment of these skin disorders. The inflammatory process is intricate and involves several key steps: recognition of pathogens or damage, activation of signaling pathways (nuclear factor-kappa B (NF-κB) and activator protein-1 (AP-1)), release of pro-inflammatory mediators (ILs and TNF-α), vasodilation, increased permeability, migration of immune cells, and phagocytosis [38]. The inflammatory properties of DME have been demonstrated through in vitro and in vivo assays. DME has been shown to significantly and dose-dependently reduce the production of PGE2 and NO and inhibit protein and mRNA expression in COX-2 and iNOS activities [24]. It can also modulate signaling pathways such as NF-κB and MAPK [24,39]. DME effectively inhibits the activity of inflammatory mediators, such as NO, TNF-α, and IL-6, at doses of 200 and 400 μg/mL [15]. In vivo assays have indicated that DME can reduce the levels of pro-inflammatory cytokines (TNF-α, IL-2, IL-4, IL-5, IL-6, IL-10, IL-12, IL12, IL-13, and IFN-γ) in immunized BALB/C mice at high concentrations (125, 250, and 500 mg/kg) [40]. Another group reports that DME markedly inhibits inflammatory cytokines and TGF-β1 expression in diabetic Sprague–Dawley rats when administered at 25 mg/kg [41,42]. In this study, a meta-analysis was designed to compare the expression of these pro-inflammatory mediators (ILs and TNF-α) and NO production between the DME treatment and the control groups. The results showed that DMEs effectively suppressed the expression of IL mediators and NO production compared to the control groups. These findings suggest that DMEs show a potential as anti-inflammation agents.

Microbial infections significantly affect the development and progression of various skin disorders. The skin is a protective barrier against external pathogens; however, when this barrier is weakened, microorganisms (e.g., bacteria, viruses, fungi, and parasites) can penetrate the skin, causing infections [43]. There are several instances of an association between microbial infections and skin disorders. Skin infections caused by *S. aureus* or *Streptococcus pyogenes* (*S. pyogenes*) can result in red sores, blisters, redness, swelling, or pain. *Candidiasis*, a yeast infection caused by *Candida* species, generally affects areas with skin folds and can cause diaper rash, thrush, and intertrigo [44]. *Propionibacterium acnes* contributes to inflammation and colonizes hair follicles, leading to acne [45]. *Bacillus oleronius*, *Bacillus simplex*, *Bacillus pumilus*, and *Bacillus cereus*, found in *Demodex folliculorum*, commonly inhabit human hair follicles [46]. They have been identified as inflammation triggers in skin conditions such as rosacea and psoriasis [46]. Bacterial infections can also cause atopic dermatitis by breaking the skin barrier. Therefore, effectively managing skin disorders often involves addressing the underlying microbial infection, which may require topical or systemic antimicrobial agents. DMEs exhibit a wide range of compounds with antimicrobial properties. Phenolic, flavonoid, and terpenoid compounds significantly disrupt microbial cell membranes, inhibit enzymatic activity, and interfere with cellular processes [4,17]. Alkaloids are nitrogen-containing compounds with antimicrobial properties that can disrupt microbial cell membranes, inhibit protein synthesis, and interfere with DNA replication [47]. Saponins, which have detergent-like properties, can disrupt microbial cell membranes, leading to cell lysis [48]. Indeed, DME exhibits high antimicrobial activity against *S. mutans* and *C. albicans* at concentrations of 40, 80, and 100 μg/mL [4]. Additionally, DME can inhibit the growth of *P. acnes*, *P. ovale*, and *Malassezia furfur* (*M. furtur*) with an MIC of 17.3%, 21.6%, and 15.7%, respectively [12]. Another study reports that DME shows antimicrobial effects at a high concentration (>5 mg/disc), resulting in a clear zone of 8.63 ± 0.31 mm for *S. aureus* and 8.17 ± 0.49 mm for *S. epidermidis* [16]. A meta-analysis confirmed that DMEs significantly inhibited the growth of bacteria that cause skin disorders with a high statistical significance (I^2^ = 31%, *p* = 0.21). DMEs are promising antimicrobial ingredients for cosmetic applications, particularly for treating red sores, blisters, and redness.

A meta-analysis revealed that DMEs show positive effects for nearly all relevant perspectives, including high biocompatibility, suppression of melanin synthesis and tyrosinase production, inhibition of pro-inflammatory markers and NO production, and suppression of bacteria growth.

## 6. Limitations of Study

This meta-analysis has identified three main limitations. First, this study includes a small number of studies, which may lead to a low reliability of analysis results. Second, because *D. morbifera* is a medicinal plant cultivated solely in Korea, its statistical significance could not be determined from a geographical perspective, resulting in a low statistical significance worldwide. Finally, all studies were conducted in vitro, which may limit the analytical diversity based on the study design type. Further analyses should be conducted when in vivo studies and clinical trials of DME-based treatments for skin conditions are available.

## 7. Conclusions

This systematic review and meta-analysis found that DMEs effectively control the symptoms of some skin disorders caused by hyperpigmentation, inflammation, and microbes. The results showed that DMEs showed beneficial effects in inhibiting melanin synthesis, reducing the expression of IL markers and NO production, and suppressing the development of bacteria, compared to the control groups. The meta-analysis achieved a good statistical significance without heterogeneity when comparing the mean differences between the two groups. However, since the meta-analysis was based on in vitro studies, the results may not present a high statistical significance for overall cosmetic applications. Therefore, further meta-analyses should be conducted when in vivo and clinical trial databases become available. Moreover, since the *D. morbifera* plant is found mainly in East Asia, especially Korea, its worldwide application is still limited. However, the *Dendropanax* genus has many species worldwide, and this study may provide good evidence for further investigations into the treatment efficiency of other *Dendropanax* species for skin disorders. In conclusion, with current in vitro evidence, the application of *D. morbifera* and other *Dendropanax* species to skin disorder treatment can attract the attention of scientists. Subsequently, literature databases can be enriched and provide a good premise for further applications.

## Figures and Tables

**Table 1:** Summary of the characteristics of included studies.

References	Region	Raw *D. morbifera*	Extraction Methods	Physicochemical Properties		Cosmetic Properties	Experimental Subjects	Main Outcomes
				Compounds	Mean ± SD			
Akram et al., 2016 [24]	Korea	Leaves	Methanol extraction	—	—	Anti-inflammatory	RAW264.7 cells	Inhibition of the expression of IL and TNF- α markers (pg/mL) and NO production (%)
Choi et al., 2021 [26]	Korea	Leaves	Ethanol extraction	—	—	Antimicrobial	HFDPC cells *S. aureus*	Inhibition of bacterial growth (mm) Cell viability (%)
Choo et al., 2019 [15]	Korea	Leaves	Ethanol extraction	—	—	Anti-inflammatory	RAW246.7 cell	Inhibition of the expression of IL and TNF- α markers (pg/mL) and NO production (%)
Hoang et al., 2022 [13]	Korea	Leaves and wood	Microwave-assisted extraction	Phenolic (mg GAE/ug) Flavonoid (mg QE/g)	313.03 ± 3.9 32.37 ± 0.9	Whitening effect Biocompatibility	HaCaT cells	Inhibition of tyrosinase production (%) Cell viability (%)
Jang et al., 2020 [16]	Korea	Leaves	Ethanol extraction	Phenolic (mg GAE/g) Flavonoid (mg QE/g)	48.77 ± 1.52 31.86 ± 1.44	Antimicrobial	L929 cells *S. aureus*	Inhibition of bacterial growth (mm)
Kim et al., 2015 [4]	Korea	Branches	Ethanol extraction	—	—	Antimicrobial Biocompatibility	HaCaT cells *S. mutans*	Inhibition of bacterial growth (mm) Cell viability (%)
Kim et al., 2021 [3]	Korea	Leaves and stems	Methanol extraction	Phenolic (mg GAE/g) Flavonoid (mg QE/g)	51.4 ± 1.32 11.3 ± 2.01	Whitening effect	—	Inhibition of tyrosinase and melanin production (%)
Lee et al., 2015 [5]	Korea	Leaves	n-hexane extraction	—	—	Whitening effect	B16F10 cells	Inhibition of tyrosinase production (%)
Lee et al., 2019 [19]	Korea	Leaves and branches	Hot-water extraction	—	—	Antimicrobial	*S. aureus*	Inhibition of bacterial growth (mm)
Mo et al., 2013 [27]	Korea	Leaves	Methanol extract	Flavonoids (mg RE/g)	98.53 ± 4.09	Whitening effects	B16F10 cells	Inhibition of tyrosinase and melanin production (%)
Park et al., 2013 [14]	Korea	Leaves	Ethanol extraction	—	—	Whitening effect Biocompatibility	HaCaT cells	Inhibition of melanin production (%) Cell viability (%)
Park et al., 2014 [28]	Korea	Leaves	Ethanol extraction	—	—	Whitening effect	B16F10 cells	Inhibition of tyrosinase production (%)
Park et al., 2016 [12]	Korea	Leaves	Methanol extraction	Phenolic (mg GAE/g) Flavonoid (mg QE/g)	74.08 ± 7.18 97.36 ± 2.24	Anti-inflammatory Biocompatibility	HFDPC cells *P. acnes*	Inhibition of bacterial growth (mm) Cell viability (%)
Park et al., 2017 [25]	Korea	Leaves	Water extraction	—	—	Anti-inflammatory	EL-4 T cells	Inhibition of the expression of IL markers (pg/mL)
Park et al., 2019 [29]	Korea	Leaves and branches	Ethanol extraction	—	—	Biocompatibility	HFDPC cells	Cell viability (%)
Park et al., 2020 [21]	Korea	Leaves	Water extraction	—	—	Whitening effect	B16F10 cells	Inhibition of tyrosinase and melanin production (%)

**Table 2:** Risk of bias rating in individual studies.

Study	Random Sequence Generation	Allocation Concealment	Selective Reporting	Blinding of Participants	Blinding of Outcome Assessment	Incomplete Outcome Data
Akram et al., 2016 [24]						
Choi et al., 2021 [26]						
Choo et al., 2019 [15]						
Hoang et al., 2022 [13]						
Jang et al., 2020 [16]						
Kim et al., 2015 [4]						
Kim et al., 2021 [3]						
Lee et al., 2015 [5]						
Lee et al., 2019 [19]						
Mo et al., 2013 [27]						
Park et al., 2013 [14]						
Park et al., 2014 [28]						
Park et al., 2016 [12]						
Park et al., 2017 [25]						
Park et al., 2019 [29]						
Park et al., 2020 [21]						

Risk of bias rating: 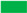 Low risk of bias; 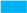 High risk of bias; 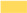 Uncertain.

**Table 3:** Summary of standardized mean differences (SMDs) comparison between the DME treatment and control groups.

Cosmetic Properties	No. of Studies	Comparison Perspectives	n	SMD (95% CI)	I^2^ (%)
Biocompatibility	5	Cell viability of HaCaT cells (%)	12	0.73 [−0.34, 1.80]	46
		Cell viability of HFDPC cells (%)	10	1.15 [−0.09, 2.39]	49
Whitening effects	7	Inhibition of the production of tyrosinase (%)	21	6.85 [4.27, 9.44]	33
		Inhibition of the production of melanin (%)	11	23.38 [12.94, 33.82]	0
Anti-inflammatory properties	4	Inhibition of the expression of IL markers (pg/mL)	18	5.22 [3.12, 7.33]	29
		Inhibition of the expression of TNF-α (pg/mL)	7	1.09 [−0.5, 2.67]	83
		Inhibition of the production of NO (%)	7	6.92 [2.89, 10.96]	0
Antimicrobial properties	5	Inhibition of the growth of bacteria (mm)	18	[3.15, 7.42]	31

## Data Availability

Not applicable.
